# The Acceleration of Diabetic Wound Healing by Low-Intensity Extracorporeal Shockwave Involves in the GSK-3β Pathway

**DOI:** 10.3390/biomedicines9010021

**Published:** 2020-12-30

**Authors:** Rong-Fu Chen, Yun-Nan Lin, Keng-Fan Liu, Chun-Ting Wang, Savitha Ramachandran, Ching-Jen Wang, Yur-Ren Kuo

**Affiliations:** 1 Division of Plastic Surgery, Department of Surgery, Kaohsiung Medical University Hospital, Kaohsiung 807, Taiwan; dr.chenrf@gmail.com (R.-F.C.); yunnan1123@gmail.com (Y.-N.L.); cell77821@gmail.com (K.-F.L.); chuntingb20120@gmail.com (C.-T.W.); 2 Department of Plastic and Reconstructive Surgery, KK Women’s and Children’s Hospital, Singapore 229899, Singapore; savitha.ramachandran@singhealth.com.sg; 3 Department of Orthopaedics, Kaohsiung Chang Gung Memorial Hospital, Kaohsiung 833, Taiwan; cjwang1211@gmail.com; 4 Faculty of Medicine, College of Medicine, Orthopaedic Research Center, Regenerative Medicine and Cell Therapy Research Center, Kaohsiung Medical University, Kaohsiung 807, Taiwan; 5 Department of Biological Sciences, National Sun Yat-Sen University, Kaohsiung 804, Taiwan; 6 Academic Clinical Programme for Musculoskeletal Sciences, Duke-NUS Graduate Medical School, Singapore 169857, Singapore

**Keywords:** diabetic wound healing, GSK-3β, β-catenin, BIO, Wnt, extracorporeal shockwave therapy

## Abstract

Previous studies have demonstrated that extracorporeal shock wave therapy (ESWT) could accelerate diabetic wound healing and that the inhibition of glycogen synthase kinase-3β (GSK-3β) is involved in epithelial differentiation during wound healing. This study investigated whether the enhancement of diabetic wound healing by ESWT is associated with the GSK-3β-mediated Wnt/β-catenin signaling pathway. A dorsal skin wounding defect model using streptozotocin-induced diabetic rodents was established. Rats were divided into 4 groups: group 1, normal controls without diabetes; group 2, diabetic controls without treatment; group 3, diabetic rats receiving ESWT; and group 4, rats receiving 6-bromoindirubin-3′oxime (BIO), a GSK-3β inhibitor, to trigger Wnt/β-catenin signaling. Tissue samples were collected and analyzed by immunohistochemical (IHC) staining and quantitative RT-PCR. The ESWT and BIO-treated groups both exhibited significant promotion of wound healing compared to the healing in controls without treatment. RT-PCR analysis of Wnt-1, -3a, -4, -5a, and -10 and β-catenin expression showed significantly increased expression in the ESWT group. The IHC staining showed that Wnt-3a and -5a and β-catenin levels were significantly increased in the ESWT and BIO treatment groups compared to the control groups. ESWT enhancement of diabetic wound healing is associated with modulation of the GSK-3β-mediated Wnt/β-catenin signaling pathway.

## 1. Introduction

Diabetic wounds occur commonly and reduce the quality of life of those affected; furthermore, they represent a clinical and socioeconomic burden in health care worldwide [1]. Appropriate wound treatments include aggressive debridement, infection control, pressure offloading, and modern wound dressing [2]. Numerous therapeutic approaches have been developed to improve wound healing, such as hyperbaric oxygen therapy and negative pressure wound therapy [3]. Although these therapeutic options are useful adjuncts to standard treatment, some treatment outcomes remain unclear [4,5,6].

Extracorporeal shockwave therapy (ESWT) acts as a physical stimulus that promotes biological healing processes through mechanotransduction. The biological effects of ESWT are reported such as wound healing, angiogenesis, bone remodeling, and tissue regeneration [7]. ESWT has been applied in different clinical fields and yielded encouraging results regarding tissue regeneration [8,9,10]. Studies also have shown that specific cellular molecules include Wnt, ATP/P2X7, focal adhesion kinase (FAK), VEGF, brain-derived neurotrophic factor (BDNF), extracellular-signal-regulated kinase (ERK), and protein kinase R-like endoplasmic reticulum kinase/activated transcription factor (PERK/ATF) were modulated by ESWT [11]. Our previous animal study showed that ESWT could enhance wound healing and salvage ischemic tissue through increasing topical circulation, suppressing inflammatory responses, inducing cell proliferation, and accelerating neovascularization through enhancing endothelial nitric oxide synthase (eNOS) and vascular endothelial growth factor (VEGF) [12,13,14,15]. Besides, our clinical trials revealed that ESWT can accelerate wound healing and neoangiogenesis in patients with diabetes [16,17]. However, mechanisms underlying the effects of ESWT on the wound healing process have not been fully elucidated. 

Glycogen Synthase Kinase-3 (GSK-3) is a serine/threonine kinase that is ubiquitously expressed as a key regulator involved in various cellular events and signaling pathways, such as the insulin pathway and Wnt/β-catenin signaling pathways, etc. [18,19]. Studies have indicated that Wnt/β-catenin signaling plays a role in epithelial differentiation during cutaneous wound healing [20,21]. The Wnt family of genes in mammals has been found to comprise 19 genes that bind to Frizzled receptors on the surface of specific cells and are classified into two groups according to whether they activate the canonical (Wnt/β-catenin; Wnt1, 2, 3, 8, and 10) or noncanonical (β-catenin-independent; Wnt4, 5, 6, 7, and 11) signaling pathways [22,23]. Activation of the canonical Wnt pathway through the inhibition of GSK-3β activity results in the nuclear accumulation of unphosphorylated β-catenin to activate target genes [24]. However, noncanonical Wnt pathways are β-catenin independent and involved in the Wnt-atypical protein kinase C pathways [25]. Studies have demonstrated that Wnt pathways play important roles in tissue morphogenesis, bone metabolism, and epithelial cell migration [26,27,28,29]. However, whether GSK-3 phosphorylation-mediated Wnt signaling contributes to the wound healing effect of ESWT remains unclear.

Therefore, in the present study, we extended our previous studies and investigated whether the promoting effect of ESWT on wound healing is involved in Wnt/β-catenin related pathway by using dorsal wounding in streptozotocin (STZ)-induced diabetic rats [14,30]. 6-bromoindirubin-3’-oxime (BIO), a selective inhibitor of GSK-3 activity that triggers Wnt/β-catenin signaling, was applied to evaluate the wound healing process [31]. Quantitative RT-PCR and IHC staining of peri-wound tissue was performed to elucidate the biosignals between ESWT- and BIO-treated groups during wound healing.

## 2. Experimental Section

### 2.1. Animal Model

The animal models were the same ones used in our previous studies [13,14,32]. All the animals were cared for humanely according to the Guide for the Care and Use of Laboratory Animals provided by the National Institute of Health. The housing, care conditions of the animals and all experimental procedures are approved and monitored followed the regulations of the Institutional Animal Care and Use Committee (IACUC Animal use protocol approval number: 2007111902). The wounding operation was performed four weeks after the STZ injection of four-month-old male Wistar rats. Following former methods, rats received an intraperitoneal injection of freshly prepared STZ (Sigma-Aldrich, St Louis, MO, USA) solution at a dose of 50 mg/kg body weight [30,33]. A dorsal skin wounding defect with an area of 6 × 5 cm^2^ involving entire skin excision below the level of the dorsal fascia was used as the wounding defect model [14]. The wound margin was sutured in place with 4-0 silk sutures to prevent wound contracture. The sutured wound was then temporarily covered with transparent Tegaderm (3M HealthCare, Borken, Germany) before treatment.

### 2.2. Experimental Design

Isoflurane was used for inhalational general anesthesia, and intramuscular injection of atropine (0.1 mg/kg) was performed to prevent excessive secretion of saliva during the operation. Forty four-month-old male Wistar rats were divided into four groups (subgroup *n* = 10). In group 1 (normal control, NC group), the dorsal skin defect was created, and no treatment was applied. In group 2 (diabetic control, C group), diabetic rats were employed and not treated. In group 3 (ESWT), diabetic rats were treated with ESWT (800 impulses at 10 kV, MTS Reflector Type CP155, Konstanz, Germany) at a dosage equivalent to an energy density of 0.09 mJ/mm^2^ (low-intensity) in two sessions along the wound margin (100 impulses/area × 8 areas in all wound edges) on postoperative days 3 and 7. This dosage and timing followed our previous study protocol [14]. In group 4, diabetic rats were treated with 200 μg/kg BIO (Merck KGaA, Darmstadt, Germany) three times per week for four weeks [34].

### 2.3. Estimation of the Wound Healing Area

The area of wound healing was calculated once a week after the operation using the template technique as previously reported [13,32]. The traced area was cut, measured, and calculated by the formula (1 − A1/A0) × 100%, where A0 is the original wound area (6 × 5 cm^2^) and A1 is the unhealed area. The area was calculated once a week until the whole wound had healed [13].

### 2.4. Quantitative Real-Time RT-PCR

Total RNA was extracted and purified from peri-wound tissue biopsies using QIAzol reagent (Qiagen, Valencia, CA, USA) following the manufacturer’s protocols on days 3 and 10 post-ESWT and at similar time points in the BIO-treated and control groups. One microgram of total RNA was initially reverse-transcribed into cDNA. A PCR mixture (25 μL) containing cDNA template, 2.5 μM each of sense and antisense primer, and 2× iQ SYBR Green Supermix was processed using the iCycler Real-time PCR Detection System (Bio-Rad Laboratories, Hercules, CA) with denaturation at 95 °C for 5 min followed by 40 cycles at 94 °C for 15 s, 52 °C for 20 s, and 72 °C for 30 s for PCR amplification [15,24]. The difference of threshold cycle (ΔCt) was calculated for the target genes and β-actin of each sample. The relative expression in each treatment group was defined as the fold change compared with the control group and was calculated as 2^−ΔΔCt^, where ΔΔCt = ΔCt_treatment_ − ΔCt_control_. The primer sequences are available upon request.

### 2.5. Immunohistochemical (IHC) Staining and Histomorphometric Examination

Semiquantitative IHC staining was performed by using a horseradish peroxidase-diaminobenzidine (HRP-DAB) staining kit (R&D Inc., Minneapolis, MN, USA) following our previous publications [14,30]. Biopsy tissues were collected from the wound margin on days 3 and 10 following the final treatment of the ESWT group and at corresponding time points in the BIO and control groups. Polyclonal antibodies against Wnt3a, Wnt5a, and β-catenin (Santa Cruz, Santa Cruz, CA, USA) were employed as the primary antibodies at 1:100 dilutions in PBS, and sections were incubated with primary antibodies for 1 h. The slides were further incubated with goat anti-rabbit biotinylated-antibodies for 30 min as per our previous publication [14]. For immunostaining quantification, four random images from each selected area were acquired at 400× magnification. All images were analyzed using the image-processing software Image-Pro Plus 6 (Media Cybernetics, Inc., Rockville, MD 20852, USA) as described in our previous publications [14,30].

### 2.6. Data management and Statistical Analysis

Experimental data are presented as the means ± standard deviation (SD). Significance levels were set at the 5% level using the Student *t*-test. Mean differences between groups were analyzed by one-way or repeated ANOVA with Tukey’s post-test or via two-way ANOVA with Duncan’s and Bonferroni’s post-tests as appropriate. Significance was evaluated at *p* < 0.05. 

## 3. Results

### 3.1. ESWT and BIO Treatment Both Accelerated Wound Healing

**Figure 1:**
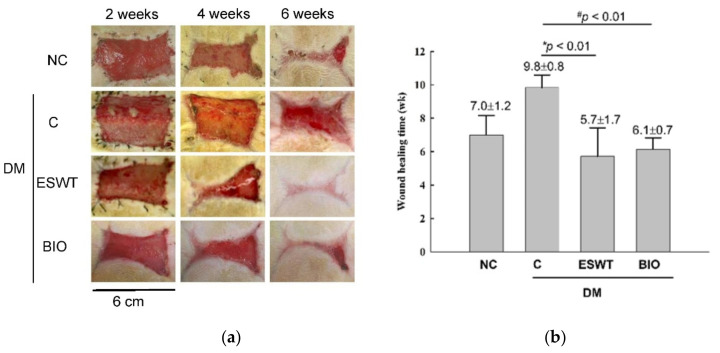
Extracorporeal shockwave therapy (ESWT) and 6-bromoindirubin-3′-oxime (BIO) both enhanced diabetic wound healing in a rodent wounding model. (**a**) This figure shows the healing time course observed following ESWT and BIO treatment at 2, 4, and 6 weeks. Wound size was significantly reduced in the ESWT and BIO groups compared with the diabetic control (C) group. NC, normal control. (**b**) Wound healing time was significantly decreased in ESWT-treated and BIO-treated diabetic rats compared with diabetic control (C) rats (* *p* < 0.01, # *p* < 0.01). Subgroup *n* = 10.

The in vivo results revealed that the size of the wound margin was significantly decreased in the ESWT- and BIO-treated diabetic groups compared with the diabetic control group (Figure 1a). The time to complete wound healing was significantly reduced in the ESWT group compared to the diabetic control group (Figure 1b, 5.7 ± 1.7 weeks versus 9.8 ± 0.8 weeks, *p* < 0.01). The time of wound healing was also significantly decreased in the rats receiving BIO treatment compared with those in the diabetic control group (6.1 ± 0.7 weeks versus 9.8 ± 0.8 weeks, *p* < 0.01). However, there was no significant difference between the BIO and ESWT groups. The results indicated that both ESWT and BIO enhanced diabetic wound healing.

### 3.2. ESWT Enhancement of Wound Healing Is Associated with the Expression of Genes in the Canonical and Noncanonical Wnt/β-Catenin Pathways

**Figure 2:**
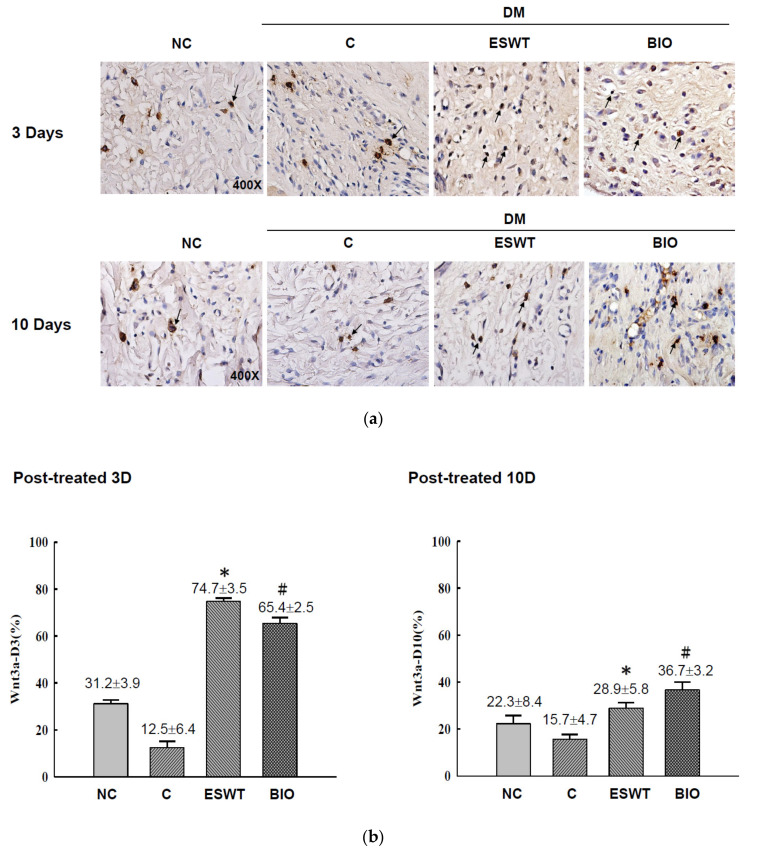
Extracorporeal shockwave therapy (ESWT) increased Wnt3a expression as revealed by IHC staining. (**a**) Wnt3a expression levels were determined at the junction zone of the wound edge on days 3 and 10 after ESWT and BIO. (**b**) Wnt3a expression was significantly increased, especially in the fibroblasts in the basal epidermal and subcutaneous layers, in the rats subjected to ESWT and BIO treatment compared with control rats. This indicated signals were statistically significant (* *p* < 0.001, # *p* < 0.01). Magnification, 400×.

**Figure 3:**
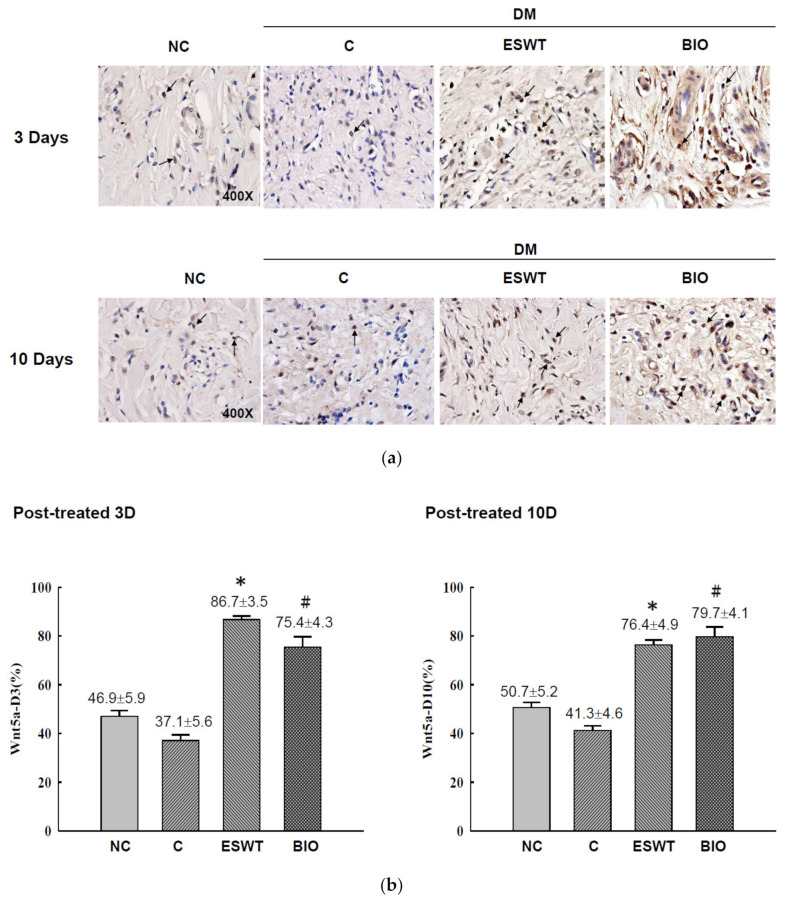
Extracorporeal shockwave therapy (ESWT) increases Wnt5a expression as revealed by IHC staining. (**a**) Wnt5a expression levels were determined at the junction zone of the wound edge on days 3 and 10 after ESWT and BIO. (**b**) Wnt5a expression levels were significantly increased, especially in the fibroblasts in the basal epidermal and subcutaneous layers, in the rats subjected to ESWT and BIO compared with the rats in the diabetic control (C) group. The indicated signals were statistically significant (* *p* < 0.001, # *p* < 0.01). DM, diabetes mellitus rats. Magnification, 400×.

**Figure 4:**
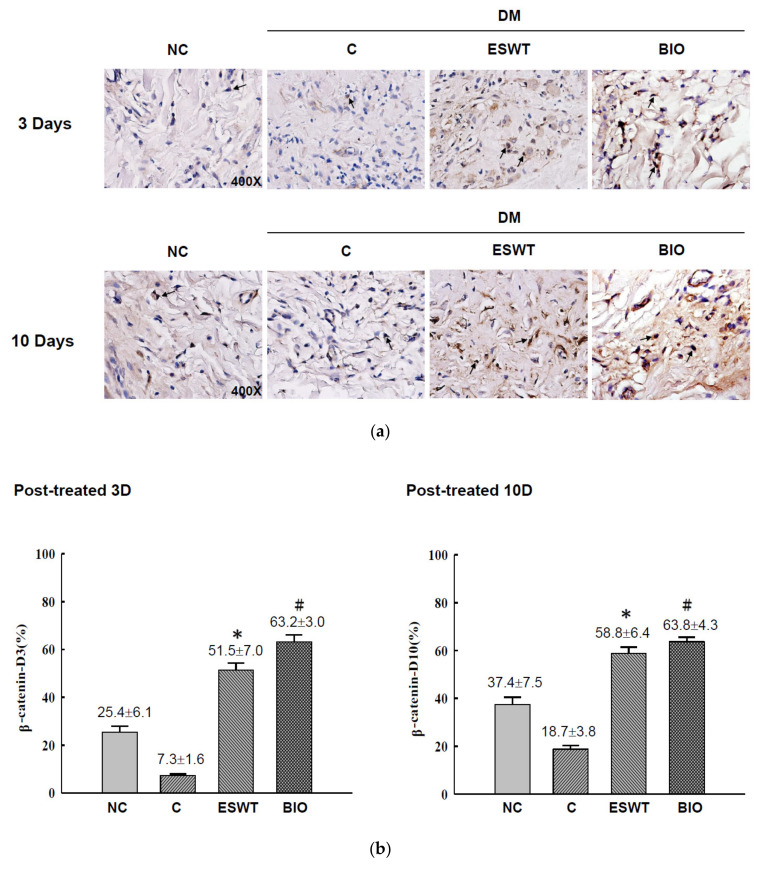
Extracorporeal shockwave therapy (ESWT) increases β-catenin expression as revealed by IHC staining. (**a**) β-catenin expression levels were determined at the junction zone of the wound edge on days 3 and 10 after ESWT and BIO. (**b**) β-catenin expression levels were significantly increased, especially in the fibroblasts in the basal epidermal and subcutaneous layers, in the rats subjected to ESWT and BIO compared with the rats in the diabetic control (C) group. The indicated signals were statistically significant (* *p* < 0.001, # *p* < 0.01). DM, diabetes mellitus rats. Magnification, 400×.

To study the effects of ESWT on Wnt protein and β-catenin expression during wound healing in vivo, tissue biopsies were retrieved from the wound edges of all groups. The expression levels of Wnt3a, Wnt5a, and β-catenin in cutaneous tissues on days 3 and days 10 after ESWT and BIO treatment were measured. Wnt3a expression was significantly higher in ESWT (74.7%) than in NC (31.2%), C (12.5%), or BIO (65.4%) on day 3, as evidenced by IHC staining, shown in Figure 2b (IHC staining in Figure 2a). However, Wnt3a expression after ESWT and BIO had decreased to 28.9% and 36.7 %, respectively, on day 10. In contrast, Wnt5a expression was higher after ESWT (86.7%) and BIO treatment (75.4%) than in control rats (37.1%) on day 3 (Figure 3b; IHC staining in Figure 3a). Interestingly, Wnt5a expression in the wound edge was persistently high after ESWT (76.4%) and BIO (79.7%) treatment and higher in those treatments than that in Control (41.3%) on day 10. The expression level of β-catenin was 51.5% to 58.8% in the ESWT group and 63.2% to 63.8% in the BIO group on day 3 and day 10, respectively (Figure 4b; IHC staining in Figure 4a). These results indicated that ESWT enhances diabetic wound healing via both the canonical and noncanonical Wnt/β-catenin pathways.

### 3.3. ESWT Increased the mRNA Expression of Wnt and β-Catenin Genes in Peri-Wounding Tissue

To investigate the expression of genes involved in Wnt/β-catenin signaling during wound healing, total RNA was harvested from peri-wounding tissue from rats in the normal control group (NC), diabetic control group (C), and ESWT-treated group (ESWT). Quantitative real-time RT-PCR was performed to assess the mRNA levels of Wnt1, 3a, 4, 5a, and 10 and β-catenin on day 3 after ESWT (Table 1). The expression of Wnt1, 3a, 4, and 5a and β-catenin were significantly increased by approximately two to eightfold in the ESWT group compared with the NC and C groups. Wnt10 had an increasing trend, but the differences were not significant. These results demonstrated that ESWT enhanced diabetic wound healing and that wound healing was associated with the Wnt/β-catenin signaling pathway.

## 4. Discussion

Numerous studies have revealed that ESWT represents a potential therapeutic approach for tissue repair and regeneration [35,36,37,38]. Clinical trials have revealed beneficial effects of ESWT in treating chronic diabetic foot ulcers, bone union, tendinitis, spine fusion, and ischemic heart disease [9,39]. Although studies have demonstrated that ESWT improves tissue regeneration by promoting angiogenesis, increasing cell proliferation, reducing apoptosis, enhancing extracellular matrix metabolism, and reducing the inflammatory response, the mechanisms underlying its effects on the wound healing process have not been well elucidated [10,37,40].

GSK-3 is a serine/threonine kinase that is ubiquitously expressed and regarded as a regulator of various cellular events and signaling pathways, such as the insulin pathway and Wnt/β-catenin signaling pathways. Studies have discovered multifaceted roles of GSK-3 in Wnt signal transduction. Recent data indicate that GSK-3 plays an important role in the Wnt cascade by phosphorylating the Wnt receptors on low-density lipoprotein receptor-related proteins (LRP5/6), thereby causing suppression of GSK-3 activity by Wnt/β-catenin stabilization [41]. Another Wnt receptor for signal transduction is Frizzled that recruits the cytosolic protein Dishevelled (Dvl), which then binds to Axin and thereby recruits the destruction complex to the activated receptor complex [42]. The Dvl-Axin interaction is essential for Wnt/β-catenin signal transduction, it is worthy of further research and investigation. Our previous study showed that ESWT accelerates tissue regeneration in an osteoarthritic knee model in association with the expression of Wnt5a and β-catenin [43]. In this study, we investigated whether ESWT promotes diabetic wound healing in association with GSK-3/β-catenin phosphorylation signaling. BIO is a specific pharmacological inhibitor of GSK-3 that actives the β-catenin cascade [31]. Our results revealed that both the ESWT and BIO groups had similar accelerated wound healing rates compared to those in the untreated-diabetes control groups [13]. This finding indicated that GSK-3/β-catenin signaling is likely involved in the ESWT-induced enhancement of diabetic wound healing.

Membership in the Wnt family is established by amino acid code rather than functional properties [44]. The Wnt family comprises proteins associated with the canonical Wnt (β-catenin-dependent, Wnt1, 2, 3, 8, and 10a) pathway, which is involved in GSK-3β inhibition, and noncanonical Wnt pathways (Wnt4, 5a, 5b, 6, 7, and 11), which are characterized as β-catenin-independent pathways. In the present study, quantitative real-time RT-PCR revealed that ESWT increased the expression of Wnt1, 3a, 4, and 5a and β-catenin in the peri-wound area of diabetic rats at day 3 post surgery (Table 1).

Interestingly, Wnt subfamily members Wnt1 and 3a are functionally defined as canonical (β-catenin-dependent) pathway proteins, whereas Wnt4 and 5a belong to noncanonical pathways (β-catenin independent). These findings indicate that ESWT can enhance the signals of both the canonical (Wnt1 and 3a) and noncanonical (Wnt4 and 5a) Wnt pathways during the early wound healing process. Wnt5a and β-catenin continued to be highly expressed until 10 days after ESWT. BIO, as a GSK-3β inhibitor, was used as a control to evaluate the β-catenin-dependent pathway. The results showed that BIO treatment produced similar effects as ESWT, increasing Wnt3a, Wnt5a, and β-catenin expression and improving the healing time of diabetic rats. However, in this study, ESWT seemed to result in a better wound healing rate than BIO treatment. This finding indicates that other factors may be involved in diabetic wound healing after shockwave treatment. The emergence of this drug family has resulted from the recognition that GSK-3s play critical roles in the progression and regression of many kidney diseases. Further studies are required to elucidate the mechanisms underlying ESWT-induced enhancement of the wound healing process. In this study, we have demonstrated ESWT enhancing diabetic wound healings is correlated with GSK-3 related pathway. Suppression of GSK-3 expressions by using BIO also showed an acceleration of diabetic wound healing. This indicated GSK-3 inhibitors could be the potential therapeutic strategy for clinical stagnant wound healing.

## 5. Conclusions

**Figure 5:**
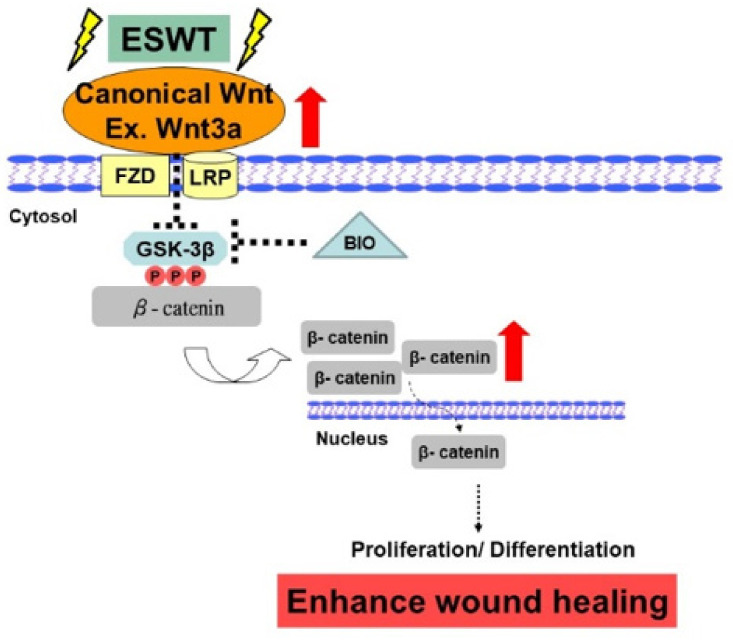
Schematic of the shockwave activation of Wnts and β-catenin-dependent pathway in wound healing. Note: dotted arrow represents its downstream or its effect; and dotted T-arrow means inhibition. The red up arrow represents increased regulation and the white flip arrow represents the translocation from the cytoplasm into the nucleus.

Our results revealed that the molecular mechanisms involved in ESWT enhancement of wound healing involve not only increased neoangiogenesis but also the GSK3β-mediated Wnt/β-catenin pathways (Figure 5). Hence, ESWT-enhanced wound healing is associated with a combination of both canonical and noncanonical Wnt ligand/coreceptors interactions as well as high levels of β-catenin signaling, resulting in enhanced cellular proliferation and differentiation during the wound healing process.

## Figures and Tables

**Table 1:** The measurement of Wnts and β-catenin expression at days 3 after ESWT by quantitative real-time RT-PCR in NC, C, and ESWT.

	NC	C	ESWT	*p*-Value
Wnt1	1.5 ± 0.9	1	7.6 ± 2.3	* p < 0.001
Wnt3a	2.2 ± 0.1	1	2.0 ± 0.4	* p < 0.001
Wnt4	1.2 ± 0.6	1	2.6 ± 0.5	* p < 0.001
Wnt5a	0.9 ± 0.0	1	4.6 ± 1.3	* p < 0.001
Wnt10	1.7 ± 0.5	1	1.5 ± 0.4	0.242
β-catenin	0.8 ± 0.3	1	1.8 ± 0.1	* p < 0.001

NC: normal control; C: diabetic control; ESWT: extracorporeal shockwave therapy. * Experimental results are presented as the means ± standard deviation and obtained from six specimens.
